# Telomere-to-Telomere genome assemblies of human-infecting *Encephalitozoon* species

**DOI:** 10.1186/s12864-023-09331-3

**Published:** 2023-05-04

**Authors:** Anne Caroline Mascarenhas dos Santos, Alexander Thomas Julian, Pingdong Liang, Oscar Juárez, Jean-François Pombert

**Affiliations:** Department of Biology, Illinois Institute of Technology, Chicago, IL USA

**Keywords:** *Encephalitozoon*, Telomeres, Methylation, Heterochromatin, Computational biology, Protein structure

## Abstract

**Background:**

Microsporidia are diverse spore forming, fungal-related obligate intracellular pathogens infecting a wide range of hosts. This diversity is reflected at the genome level with sizes varying by an order of magnitude, ranging from less than 3 Mb in *Encephalitozoon* species (the smallest known in eukaryotes) to more than 50 Mb in *Edhazardia* spp. As a paradigm of genome reduction in eukaryotes, the small *Encephalitozoon* genomes have attracted much attention with investigations revealing gene dense, repeat- and intron-poor genomes characterized by a thorough pruning of molecular functions no longer relevant to their obligate intracellular lifestyle. However, because no *Encephalitozoon* genome has been sequenced from telomere-to-telomere and since no methylation data is available for these species, our understanding of their overall genetic and epigenetic architectures is incomplete.

**Methods:**

In this study, we sequenced the complete genomes from telomere-to-telomere of three human-infecting *Encephalitozoon* spp. —*E. intestinalis* ATCC 50506, *E. hellem* ATCC 50604 and *E. cuniculi* ATCC 50602— using short and long read platforms and leveraged the data generated as part of the sequencing process to investigate the presence of epigenetic markers in these genomes. We also used a mixture of sequence- and structure-based computational approaches, including protein structure prediction, to help identify which *Encephalitozoon* proteins are involved in telomere maintenance, epigenetic regulation, and heterochromatin formation.

**Results:**

The *Encephalitozoon* chromosomes were found capped by TTAGG 5-mer telomeric repeats followed by telomere associated repeat elements (TAREs) flanking hypermethylated ribosomal RNA (rRNA) gene loci featuring 5-methylcytosines (5mC) and 5-hemimethylcytosines (5hmC), themselves followed by lesser methylated subtelomeres and hypomethylated chromosome cores. Strong nucleotide biases were identified between the telomeres/subtelomeres and chromosome cores with significant changes in GC/AT, GT/AC and GA/CT contents. The presence of several genes coding for proteins essential to telomere maintenance, epigenetic regulation, and heterochromatin formation was further confirmed in the *Encephalitozoon* genomes.

**Conclusion:**

Altogether, our results strongly support the subtelomeres as sites of heterochromatin formation in *Encephalitozoon* genomes and further suggest that these species might shutdown their energy-consuming ribosomal machinery while dormant as spores by silencing of the rRNA genes using both 5mC/5hmC methylation and facultative heterochromatin formation at these loci.

**Supplementary Information:**

The online version contains supplementary material available at 10.1186/s12864-023-09331-3.

## Introduction

Microsporidia form a large and diverse assemblage of spore-forming obligate intracellular pathogens related to Fungi composed of more than 1,500 species that infect a wide range of hosts, including mammals, fish, and insects, and on which they rely heavily for energy [1]. In host cells, microsporidia are often found colocalized with the host mitochondria, facilitating access to ATP [2]. The microsporidian adaption to an obligate intracellular lifestyle strongly shaped the evolution of their genomes, which are characterized by an overall small set of genes (ranging from about 2,000 to 4,000) following a selective pruning of many formerly mandatory molecular functions turned optional in this novel environment [3]. However, not all microsporidian genomes took the same evolutionary routes, with some expanding in size due in large part to the acquisition and spread of repeated elements, while others took reduction and compaction to the extreme, discarding most repeats, introns, and even reducing the size of their coding sequences [4, 5]. These opposite trends are reflected in microsporidian genome sizes, which differ by up to an order of magnitude, from more than 50 Mb in *Edhazardia aedis* to less than 3 Mb in *Encephalitozoon* spp. [3]. As both paradigms of genome reduction in eukaryotes and human-infecting pathogens, the latter species garnered much interest, with investigations of select representative *Encephalitozoon* genomes yielding strong insights about their metabolic capabilities and potential for harm [6–9]. However, because no *Encephalitozoon* genome has been sequenced from telomere-to-telomere and since little is known about their DNA methylation, our knowledge of the genetic and epigenetic architectures of these species is incomplete.

DNA methylation is a heritable and reversible epigenetic modification that helps to regulate transcriptional activity in higher eukaryotes by acting as on/off gene switches and to maintain genome integrity via its interplay with histone lysine methylation during chromatin formation [10, 11]. The most common type of DNA methylation in eukaryotes is 5-methylcytosine (5mC), in which a methyl group is transferred to the 5’ end of cytosine rings from donor S-adenosyl-L methionine (SAM) molecules [12]. This methylation can be performed anew or inherited epigenetically following DNA replication with various DNA methyltransferases (DNMTs). DNMT3A and DNT3B have been associated with de novo methylation, DNMT1 and DNMT5 have been shown to mediate the epigenetic inheritance of methylated sites after DNA replication, while DNMT2 was found to preferentially methylate RNA molecules despite a slight DNA methylation activity [13–15]. However, not all eukaryotes feature 5mC DNA methylation. The presence of 5mC methylation and of the genes coding for DNMTs has been reported as sporadic in fungal genomes with an uneven distribution between lineages [16]. For example, the human pathogen *Cryptosporidium neoformans* —a basidiomycete— lacks de novo methylases and was shown to maintain 5mC DNA methylation solely through epigenetic mechanisms [17] whereas DNA methylation is not found in the fission and budding yeasts *Schizosaccharomyces pombe* and *Saccharomyces cerevisiae* [12]. Furthermore, while present in the ascomycete *Neurospora crassa*, DNA methylation is not essential for heterochromatin formation in this species [18]. Among the common targets for silencing in eukaryotes are the large and small ribosomal RNA (rRNA) genes, and in DNA methylation-free organisms like *S. cerevisiae*, this silencing is mediated by heterochromatin formation at rRNA gene loci [19, 20].

Eukaryote chromosomes are packed into chromatin with nucleosomes containing DNA wrapped around various proteins including histones, and the density of these nucleosomes is used to distinguish between euchromatin and heterochromatin segments [21]. Whereas euchromatin is less dense and more easily accessible, heterochromatin is much more condensed and usually inhibits transcription [22]. Heterochromatin can be either constitutive or facultative, the latter usually containing genes that must be silenced at different cellular stages [23]. Heterochromatin is also present in centromeres, with centromeric heterochromatin in most organisms featuring a histone H3 variant named centromere protein A (CENP-A; [24]). In fungi, the formation and spreading of heterochromatin is mediated via the Clr4 methyltransferase complex (CLRC) composed of seven components including the cullin Cul4, the DNA damage binding protein 1 (DDB1) homolog Rik1, and the heterochromatin protein 1 homolog Swi6 [25]. This complex is loaded at target loci via two distinct pathways dependent on or independent from RNA interference (RNAi) [26]. The RNAi-dependent pathway is an RNA-induced initiation of transcriptional silencing (RITS) complex that leverages small interfering RNAs (siRNAs), the Argonaute/Dicer endoribonucleases and the RNA-dependent RNA polymerase Rdp1 to recruit the CLRC complex at target sites [27]. In contrast, in the budding yeast *S. cerevisiae* (which lacks the Dicer and Argonaute endonucleases), RNAi-independent heterochromatin formation is mediated via the silent information regulator (SIR) complex and relies on proteins known as sirtuins [28].

In this study, to delineate the genetic architecture of the sub-3 Mb *Encephalitozoon* genomes and investigate the presence or absence of DNA methylation in the genus, we sequenced the complete genomes from telomere-to-telomere of representative isolates from three human-infecting *Encephalitozoon* species using short and long read platforms. Using a mixture of sequence- and structure-based approaches, we further investigated in silico the proteins involved in telomere maintenance, DNA methylation, and heterochromatin formation in *Encephalitozoon* species.

## Results

### Structure of the *Encephalitozoon* telomeres and subtelomeres

**Fig. 1:**
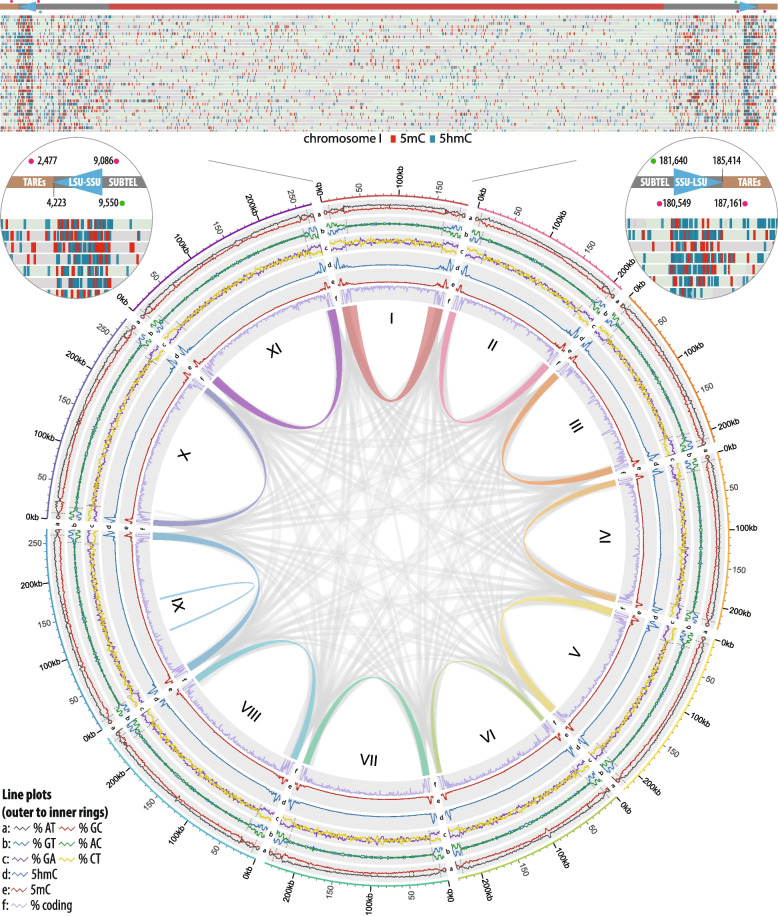
Methylation and physical maps of the *Encephalitozoon intestinalis* ATCC 50506 genome. *Top.* Distribution of 5mC and 5hmC methylated sites on the *E. intestinalis* chromosome I, as inferred from mapping of the individual nanopore sequencing reads against the genome with Megalodon (using the remora base modification model), then plotted with IGV (minimum probability: 0.8). 5mC and 5hmC methylated sites are shown in red and blue, respectively. *Small circles*. Zoom ins of the *E. intestinalis* chromosome I ends. Telomere-associated repeat elements (TAREs) and subtelomeric regions are highlighted by beige and grey lines; rRNA loci (LSU, large subunit; SSU, small subunit) are depicted with cyan triangles. Locations of 4mC and unknown base modifications (UBMs) are shown with magenta and green dots, respectively. *Circos plot*. Physical and methylation metrics of the *E. intestinalis* genome. From outer to inner concentric rings: a) AT and GC nucleotide biases (grey and red lines, respectively); b) GT and AC nucleotide biases (blue and green lines, respectively); c) GA and CT nucleotide biases (purple and yellow lines, respectively); d and e) relative proportions of 5hmC (blue) and 5mC (red) methylated sites across each chromosome; f) coding density. Shifts in nucleotide biases (rings a to c) are highlighted by dashed grey lines. Repeated loci between chromosomes (in grey) and within chromosomes (color-coded per chromosome) are highlighted by ribbons in the center of the concentric circles

The genomes of the human-infecting *Encephalitozoon* species *E. intestinalis* (ATCC 50506), *E. hellem* (ATCC 50604) and *E. cuniculi* (ATCC 50602) were sequenced from telomere-to-telomere for a total of 2,609,445, 2,707,803 and 2,847,233 bp, respectively (except for a small telomere fragment missing from *E. cuniculi* chromosome I as detailed below; Table S1)*.* This resulted in an additional 393, 456 and 350 kb of sequence compared to the largest *E. intestinalis* (ATCC 50506 [7]; 2.2 Mb), *E. hellem* (ATCC 50504 [8]; 2.25 Mb) and *E. cuniculi* (GBM1 [6]; 2.5 Mb) genome assemblies previously available in public databases. All three *Encephalitozoon* species were found to share the same pentameric telomere repeat unit (5’-TTAGG-3’) found at both ends of all chromosomes. The exact lengths of the telomere repeats were found inconsistent within and between species with the shortest and longest assemblages totaling 108 and 1,106 bp (including incomplete 5-mer repeat units), albeit this variation likely resulted from sequencing and/or assembly artefacts (Table S1). In all three *Encephalitozoon* spp., the telomeres were found flanked by telomere-associated repeat elements (TAREs; [29]) featuring two sets of tandem repeats (TARE-1 and -2) whose patterns were exclusive to each species (Table S2; Additional data S1). In *E. intestinalis*, 70-bp tandem repeats (TARE-1) adjacent to the telomeres were found immediately followed by 35-bp repeats (TARE-2) and were present in all chromosomes (Fig. 1; Table S2). A similar pattern was observed in the *E. cuniculi* chromosomes, which encompassed 37- and 59-bp TARE-1/2 repeats adjacent to the telomeres, and *E. hellem* featured a similar if less conserved organization with 65-bp TARE-1 repeats followed by 47-bp or 33-bp TARE-2 tandem repeats. The distances between the TARE-2 tandem repeats and the first/last encoded genes were found consistent within species and appears constituted from degenerated TAREs, with each chromosome featuring a shorter degenerated TAREs segment on one end (~ 3.0 to 5.2 kb) and a longer TAREs assemblage of nearly double in length (~ 5.5 to 11.4 kb) on the other end (Table S1). In all genomes, the first and last two genes of all chromosomes code for the large and small rRNA subunits, and we confirmed the total presence of 22 rRNA copies in *Encephalitozoon* genomes previously inferred from restriction mapping/fluorescence in situ hybridization (FISH) imaging [30] and from their relative sequencing depths [7, 8]. Albeit a small telomere fragment could not be assembled on *E. cuniculi* chromosome I, the adjacent locus includes the genes coding for the rRNA subunits followed by a 1,565 bp DNA segment. Considering the average length of the region between the rRNAs and telomere repeats and the length of the largest telomere repeat, we estimate that about 5 kb are missing from *E. cuniculi* chromosome I. Overall, the telomeres were found to account for approximately 125.2, 94.9 and 176.5 kb of the *E. intestinalis*, *E. hellem* and *E. cuniculi* genomes, respectively.

The telomeres in *Encephalitozoon* genomes are segregated from the remainder of the chromosomes by hypermethylated loci corresponding to the rRNA genes, which clearly mark the end of telomeres and the start of subtelomeres (Fig. 1). This hypermethylated pattern was found in all three *Encephalitozoon* genomes (Figs. 1 and S1). Epigenetic modifications in the *Encephalitozoon* genomes included 4-methylcytosines (4mC), 5-methylcytosines (5mC), 5-hemimethylcytosines (5hmC) and 6-methyladenines (6mA). In all cases, increases in 5mC and 5hmC methylation patterns overlapped with increases in nucleotide biases (described below) corresponding to the telomeres, rRNAs, and subtelomeres loci (Fig. 1). The *E. intestinalis* PacBio data also identified 5mC sites in its rRNA genes (Additional data S2) and further revealed a total of 44 4mC and 21 base modifications of unknown patterns flanking the rRNA subunits, such that two 4mC and one unknown base modification (UBM) flank each rRNA (Fig. 1). The 4mC bases flanking the rRNA-coding genes were found methylated on the same DNA strand and were distanced from one another by roughly 6,600 bp, a distance conserved across all detected instances. In all cases, the 4mC bases proximal to the telomeres were located about 1,700 bp downstream from the end of the large rRNA-coding gene while the distal ones were found located about 1,100 bp upstream from the small rRNA-coding gene. In contrast, the detected UBMs were located about 460 bp away from the closest 4mC towards the inner portion of the chromosomes, and always in the opposite DNA strand of the rRNA-coding genes.

**Fig. 2:**
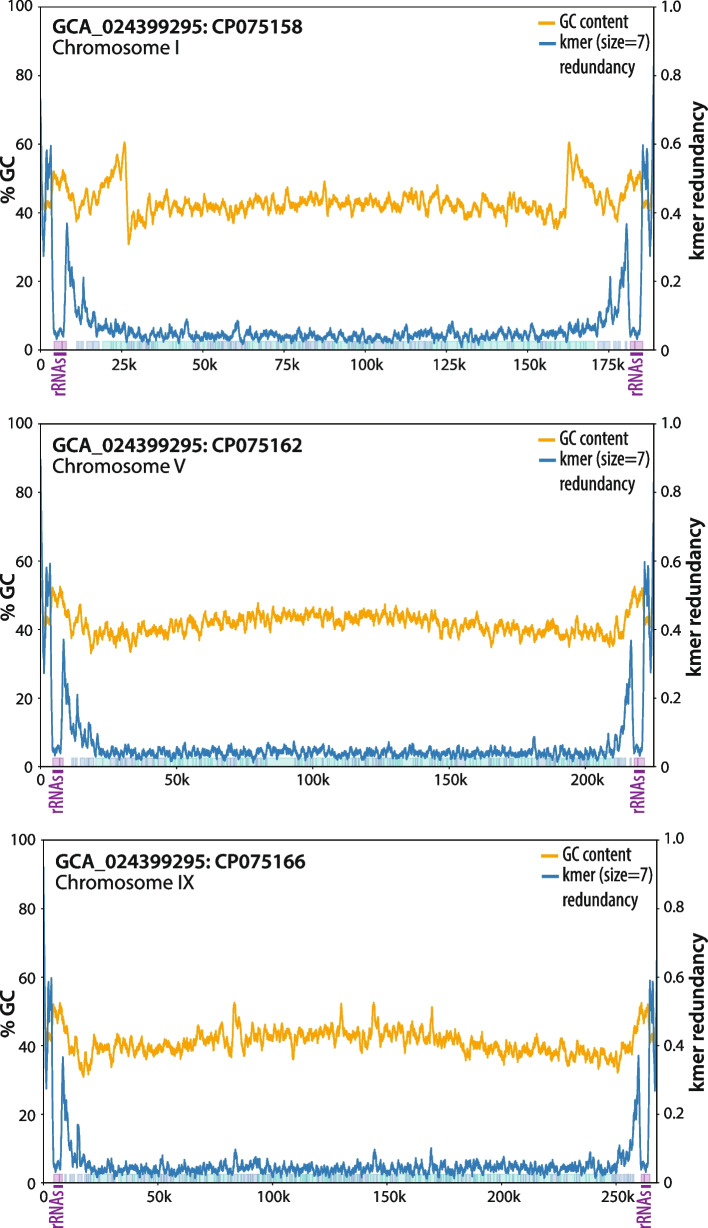
Examples of distributions of overabundant kmers in *Encephalitozoon intestinalis* chromosomes. Proportions of overabundant kmers of 7 nt across chromosomes are plotted by blue lines; GC percentages are plotted in orange, respectively. Purple, green and blue boxed highlights underneath the plots indicate locations of genes coding for rRNAs, products with known functions, and hypothetical proteins, respectively

The *Encephalitozoon* telomeres and subtelomeres displayed strong nucleotide biases compared to the chromosome cores with substantial shifts in GC/AT, GT/AC and GA/CT contents, the latter two indicating strong skews in strandedness (Fig. 1). These shifts coincided with lower coding density regions and with methylated and repeated chromosomal segments that, in all species, encompassed telomeres, rRNA genes and subtelomeres found duplicated across several chromosomes (Figs. 1 and S1). These duplicated segments were variable in size and similarity, ranging from 3.0 to 37.5 kb with a minimum sequence identity of 96.5%. For example, the longest fragment duplicated in the *E. intestinalis* genome (27.3 kb) was found near identical (99% identity) between the two ends of chromosome I and another from chromosome VIII, with shorter subsets found repeated with other chromosome ends (Figs. 1 and S2). However, except for the portion encompassing the rRNA locus, these repeated segments were not found conserved between *Encephalitozoon* species. Analyses of the *Encephalitozoon* chromosome sequences with kmers (*i.e.* substrings of length k commonly used with nucleotide sequences to identify repeats [31]) revealed that the telomeres and subtelomeres —minus the rRNA genes— both feature repetitive elements in contrast to the chromosome cores (Fig. 2).

Likewise, unlike their chromosome cores, the *Encephalitozoon* subtelomeres did not display a high level of gene order conservation, and a few recombination events involving the subtelomeres were detected between the three *Encephalitozoon* genomes (Fig. S3). Notably, based on the patterns of GC content surrounding the corresponding loci, it appears that sections of the *E. hellem* chromosomes I and VIII were recombined recently in the lineage leading this isolate, as the shifts in GC contents abutting the loci did not have time to adjust to the overall patterns observed in the *Encephalitozoon* genomes (Figs. S1 and S3). This recombination is also larger than the subtelomeres and included genes from the chromosome cores.

### Content of the *Encephalitozoon* subtelomeres

**Fig. 3:**
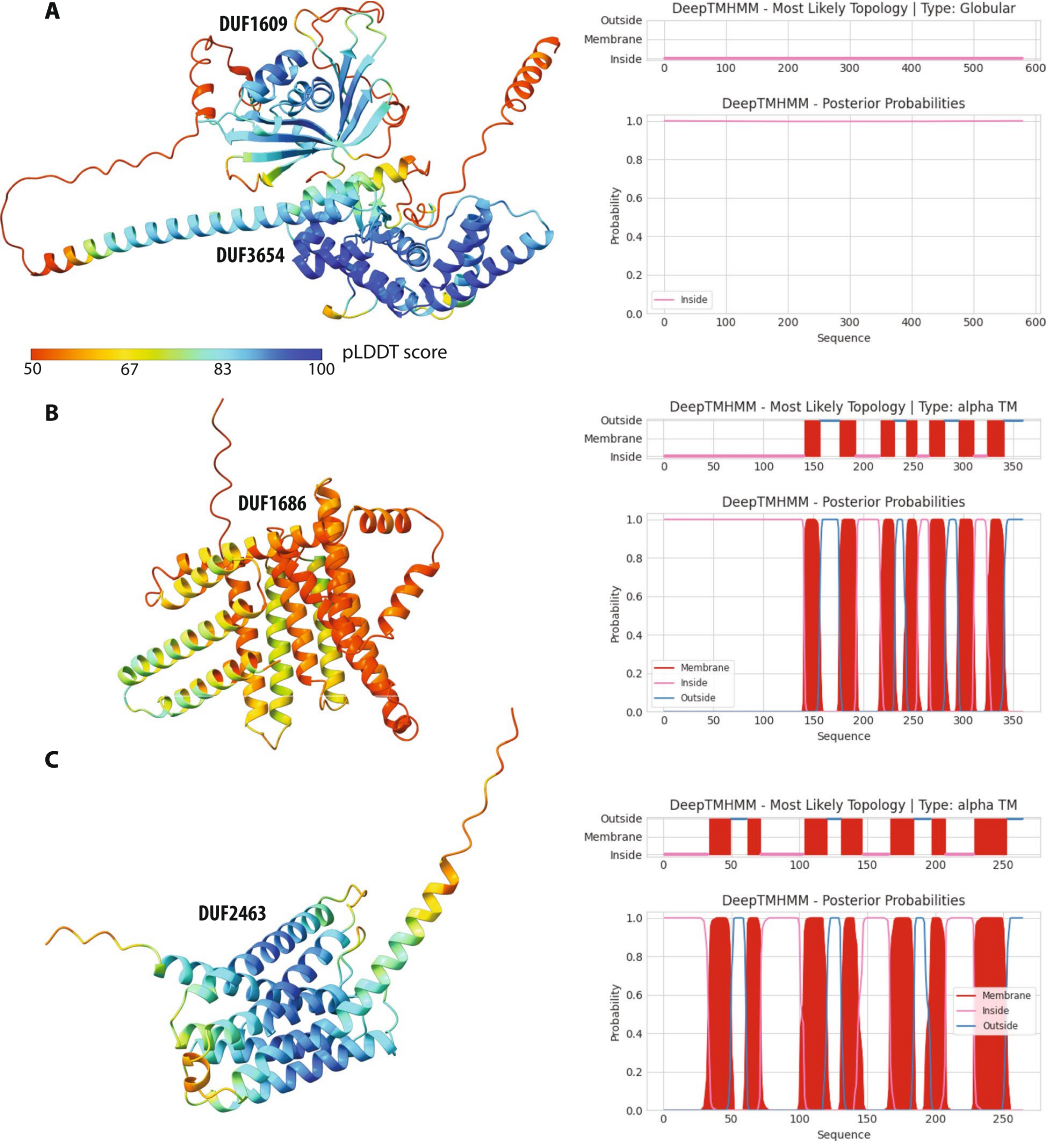
Predicted structures of *Encephalitozoon* InterAE, B, C and D proteins. **A***.* Example of a globular InterAE protein (DUF1609; aa 351–575; beta sheets) also containing an InterB domain (DUF3654; aa 179–296; alpha helices) from *E. intestinalis* (GPK93_01g00060). **B** and **C**. Examples of alpha helical InterC (DUF1686) and InterD (DUF2463) transmembrane proteins from *E. hellem* (GPU96_03g04180) and *E. intestinalis* (GPK93_02g01620), respectively. Structures are color coded by their predicted AlphaFold pLDDT confidence scores. Transmembrane domains predicted from their sequences (with DeepTMHMM) are shown on the right

Overall, the subtelomeres in *E. intestinalis*, *E. hellem*, and *E. cuniculi* totaled about 286, 432 and 418 kb and were predicted to code for 174, 327 and 417 open reading frames (ORFs), respectively (Table S1). Of these, only 34.5% (60/174) of the ORFs predicted in the *E. intestinalis* subtelomeres were found shared with both *E. hellem* and *E. cuniculi*, many of which as part of large repetitive families. *E. cuniculi* was previously shown to harbor repetitive gene families known as *interAE*, *interB*, *interC* and *interD* in its subtelomeres [9, 32], and those were found in all three *Encephalitozoon* species, albeit in different copy numbers. The InterAE, B, C and D predicted proteins accounted for a total of 50.0, 35.5 and 32.4% of the *E. intestinalis*, *E. hellem* and *E. cuniculi* predicted subtelomere ORFs, respectively, and several of the repeated InterC and InterD proteins appeared unique to *E. hellem* and *E. intestinalis*, suggesting that their genes were duplicated post-speciation (Table S3). Albeit putative functions could not be inferred for these proteins, predictive folding revealed that members of the InterC and InterD families harbor alpha-helical structures common to transmembrane proteins, congruent with previous observations [9], and corroborated by deep-learning inferences based on sequence data (Fig. 3). In contrast, members from the InterAE and InterB families were predicted to adopt globular structures. In addition to the InterAE-D proteins, the three *Encephalitozoon* genomes were predicted to code for several repeated families of unknown functions, most of which are unique to each species (Table S3). Unfortunately, structures predicted for these proteins were often of poor quality with low confidence scores and did not provide reliable insights into their putative functions (Fig. S4). Other proteins of interest predicted to be encoded in the *Encephalitozoon* subtelomeres included a choline kinase (GPK93_03g03390, GPU96_03g05810) and asparagine synthases (GPK93_02g03240, GPK93_10g19400, GPU96_02g02290, GPU96_05g10110, GPU96_05g10120, GPU96_09g16880) present in *E. intestinalis* and *E. hellem* but absent from *E. cuniculi*.

### Telomere maintenance, heterochromatin formation, and DNA methylation proteins

Several telomere and heterochromatin proteins were predicted to be encoded in the three *Encephalitozoon* genomes, including many of the components required for histone H3/lysine 9 methylation (H3K9me) epigenetic regulation (Tables 1 and S4). Telomere maintenance proteins encoded in *Encephalitozoon* spp. include the telomerase reverse transcriptase (Trt1/TERT) and RNA polymerase II CTD phosphatase Ssu72 required for telomere length regulation [33], proteins Stn1 and Ten1 from the Cdc13-Stn1-Ten1 (CST) telomere capping complex [34], proteins Pot1 and Tpz1 from the shelterin complex protecting telomeres from degradation [35], and proteins Rad32 (Mre11 in *S. cerevisiae*) and Rad50 from the *S. pombe* Rad32-Rad50-Nbs1 complex, a multifunctional complex involved in G-quadruplex binding and in DNA double strand break repair [36]. Heterochromatin formation proteins encoded in the *Encephalitozoon* genomes include heterochromatin protein Swi6 (HP1 in humans), a transcriptional repressor that regulates lysine 9 methylation on histone H3 residues [37], subunits Spt16 and Pob3 from the histone chaperone FACT (FAcilitates Chromatin Transcription) required for constitutive heterochromatin formation [38], chromatin-remodeling ATPase INO80 whose associated complex regulates heterochromatin inheritance [39], the origin recognition complex protein 1 (Orp1/ORC1) essential to heterochromatin formation in humans [40], and the histone H3 lysine modification protein Clr4 (cryptic loci regulator 4), which regulates gene expression via chromatin interaction and increases spontaneous mutations rates in fungi [41]. Clr4 is an integral component of CLRC, composed of Clr4, Cul4, Rik1, Pip1 and delocalization of Swi6 (Dos) protein Dos1 in *S. pombe*, and which is required for heterochromatin formation [25]. Components of this complex are structurally analogous to the Cullin-RING ubiquitin ligase complex Cul4/DDB1/Rbx1 (Rtt101/Mms1/Hrt1 in *S. cerevisiae*) involved in DNA repair [42], and for which we previously identified several structural analogs in *Encephalitozoon cuniculi* [43]. Dos1 (also known as Raf1 in *S. pombe*) is a DDB1–CUL4-associated factor (DCAF) forming a single 7-bladed beta-propeller [44], a common repetitive protein structure with over 20 possible structural analogs in *E. cuniculi* [43]. Altogether, these results indicate that H3K9me epigenetic regulation is likely present in *Encephalitozoon* spp.

**Table 1:** Telomere maintenance and heterochromatin formation proteins in *Encephalitozoon* spp.

***Description***	***TM score*** ^**a**^	***E-value*** ^**a**^	***E. int*** GPK93_	***E. hel*** GPU96_	***E. cun*** J0A71_
Telomerase reverse transcriptase (Trt1/TERT)	0.72	2.60E-81	09g15470	09g17180	03g05450
RNA polymerase II CTD phosphatase Ssu72	0.98	1.3E-58	06g09740	06g11230	07g15540
CST complex subunit Stn1 (Stn1)	0.89	–-	03g04440	03g05290	09g20020
CST complex subunit Ten1 (Ten1)	0.84	–-	08g14420	08g15930	04g09240
Protection of telomeres protein 1 (Pot1)	0.60	5.10E-08	05g08160	05g09440	08g17880
Pot1 and Tin2-interacting protein (Tpz1)	0.71	5.90E-07	11g20990	11g22140	01g01670
DNA repair protein Rad32 (Rad32/Mre11/NBN)	0.86	1.5E-120	05g08450	05g09710	08g18140
DNA repair protein Rad50	0.40	5.1E-192	07g11320	07g12880	05g10760
Heterochromatin protein 1 (Swi6/HP1)	0.93	4.41E-11	03g03430	03g04270	09g19010
FACT complex subunit Spt16	0.42	7.1E-112	03g03720	03g04560	09g19290
FACT complex subunit Pob3/SSRP1	0.83	2.00E-61	07g11800	07g13350	05g11230
Origin recognition complex subunit 1 (Orp1/ORC1)	0.81	3.47E-31	03g04310	03g05160	09g19890
Histone-lysine N-methyltransferase Clr4/EZH2	0.80	3.90E-67	09g17040	01g01810	03g07090
Chromatin-remodeling ATPase INO80	0.49	1.1E-273	09g17220	01g01980	03g07280
Cullin Cul4	0.88	–-	06g09780	06g11270	07g15580
Cullin Cul4	0.60	1.60E-39	07g11580	07g13140	05g11020
Cullin Cul4	0.72	8.00E-10	09g17130	01g01890	03g07180
WD-40 β-propeller proteins Rik1/DDB1	0.94	–-	05g08270	05g09550	08g17990
Cleavage and polyadenylation specificity factor 1	0.90	6.20E-62	11g20100	11g21250	01g00800
Splicing factor 3b subunit 3	0.82	2.10E-18	07g12500	07g14010	05g11930
RING finger protein Pip1/Rbx1	0.76	1.82E-36	01g01100	01g01160	11g23950
RING finger protein Pip1/Rbx1	0.76	2.30E-19	07g12190	07g13725	05g11620
Sirtuin Hst4 (Hst4/Sir2/SIRT2)	0.84	5.40E-64	–-	03g04600	09g19330
Histone H2A	0.72	2.60E-22	11g21060	11g22210	01g01740
Histone H2B	0.70	9.17E-78	08g13280	08g14830	04g08120
Transcription initiation factor IID, subunit 13	0.73	5.48E-30	04g06090	04g07150	06g13460
Histone H3/CENP-A	0.94	3.13E-33	03g04760	03g05630	09g20350
Histone H3	0.94	4.80E-44	09g15640	09g17340	03g05620
Histone H4	0.92	5.94E-21	09g15630	09g17330	03g05610
Histone-like transcription factor Y subunit gamma	0.92	6.70E-40	05g07500	05g08780	08g17220
Histone-like transcription factor (putative H2A)	0.77	1.10E-11	02g02340	02g03070	10g21680
Histone-binding protein RBBP4	0.96	6.50E-27	07g11470	07g13030	05g10910
Histone acetyltransferase RTT109	0.80	2.50E-07	01g00830	01g00900	11g23680
Histone deacetylase	0.99	0.0	03g04660	03g05530	09g20250
Histone deacetylase	0.99	0.0	09g15850	09g17530	03g05890
Histone acetyltransferase (MYST-type)	0.82	3.80E-56	09g16620	09g18300	03g06560
Histone acetyltransferase (MYST-type)	0.84	7.8E-119	10g18170	10g19350	02g03550
Histone acetyltransferase GCN5 (bromodomain)	0.91	1.73E-72	10g18990	10g20160	02g04360

^a^ Best template modelling (TM) scores and *E*-values predicted from analyses summarized in Table S4; TM scores above 0.5 indicate proteins with similar structural folds [45]

Interestingly, however, sirtuin Hst4 (Sir2 in yeast), a NAD-dependent lysine 16 histone H4 (H4K16) deacetylase that negatively regulates DNA replication origins within heterochromatin [46], was found in both *E. hellem* and *E. cuniculi* but not *E. intestinalis*. In *E. hellem* and *E. cuniculi*, the gene coding for Hst4 is found in a highly collinear portion of the chromosome cores found shared with *E. intestinalis* except for the absence of this gene (Fig. S5). This gene was not relocated elsewhere in the *E. intestinalis* genome (genome-wide sequence and structural homology searches failed to retrieve any putative homolog), indicating that sirtuin Hst4 might indeed be missing from *E. intestinalis*.

Because heterochromatin formation and DNA methylation are intertwined [47] and given the methylation patterns observed for the rRNA-coding genes (Fig. 1), we searched for the presence of genes coding for proteins involved in the methylation of DNA, rRNA and tRNA in the *Encephalitozoon* genomes (Tables 2 and S4). In eukaryotes, de novo 5hmC methylation is carried out by DNA methyltransferase 3 (DNMT3), whereas 5hmC epigenetic inheritance is performed by DNMT1 (Raf2 in *S. pombe*) [47, 48] or DNMT5 [15, 49]. However, we could not identify with confidence these DNA methyltransferases in the *Encephalitozoon* proteome. Sequence-based BLAST, Pfam and CDD searches returned no hit in the *Encephalitozoon* proteome at an *E*-value cutoff of 1e-05 whereas searches using experimentally determined structures from the RCSB PDB database against the *Encephalitozoon* predicted protein structures returned a few putative matches against miscellaneous methylases (Additional data S3), including proteins matching RNA (C5-cytosine) and S-adenosyl-L-methionine-dependent methyltransferase signatures in InterProScan5 searches (IPR023267 and IPR029063, respectively). Because both C-5 cytosine-specific DNA methylases DNMT1 and DNMT3 bind to histone deacetylases and to the H3K9 histone methyltransferase [13], we further searched for possible protein binding partners between these proteins (listed in Table 1) and the *Encephalitozoon* methyltransferases (Tables 2 and S4) using Fast Fourier transform protein–protein docking simulations. However, no obvious potential match emerged from these simulations, with only one match making it above a protein–protein interaction score (PPIscore) cutoff of 10 (Additional data S4). Further expanding the search scope to all *Encephalitozoon* proteins returned more possible binding partners but no putative DNMT1 and DNMT3 candidates. Searches for orthologs to methyl-binding proteins in *Encephalitozoon* spp. also proved unsuccessful but considering that nearly all AlphaFold-EBI predicted structures of human MBD1, MBD2 and MeCP2 methyl-binding proteins harbor low confidence scores (only one averages a pLDDT score higher than 70%), orthologs in the *Encephalitozoon* proteome (if any) are likely to have been misfolded as well and thus unlikely to be found by structural homology.

**Table 2:** DNA, rRNA and tRNA methylation proteins in *Encephalitozoon* spp.

***Description***	***TM score ***^***a***^	***E-value ***^***a***^	***E. int*** GPK93_	***E. hel*** GPU96_	***E. cun*** J0A71_
rRNA SSU methyltransferase NEP1	0.89	4.40E-24	01g00390	01g00490	11g23270
rRNA SSU dimethyladenosine transferase	0.92	6.46E-98	04g05530	04g06630	06g12910
rRNA methyltransferase E/SPB1	0.42	3.8E-113	07g12140	07g13680	05g11570
rRNA m5C methyltransferase ^b^	0.94	2.20E-128	01g01080	01g01140	11g23930
rRNA m5C methyltransferase ^b^	0.82	6.70E-93	08g13270	08g14820	04g08110
rRNA m5C methyltransferase ^b^	0.58	5.40E-75	07g11010	07g12570	05g10450
tRNA (cyt(32)/gua(34)-2'-O)-methyltransferase	0.85	2.40E-72	09g16110	09g17790	03g06150
tRNA (guanine(37)-N1)-methyltransferase	0.82	3.60E-82	04g05720	04g06780	06g13090
tRNA (guanine(26)-N(2))-dimethyltransferase	0.83	1.80E-96	08g14460	08g15960	04g09280
tRNA (guanine-N(7))-methyltransferase	0.84	6.50E-69	11g21110	11g22260	01g01790
mRNA cap guanine-N7 methyltransferase	0.88	1.30E-73	10g17890	10g19080	02g03240
Nucleomethylin/rRNA processing protein 8	0.87	7.66E-38	10g18310	10g19480	02g03690
Fibrillarin-like 2'-O-methyltransferase	0.79	7.60E-109	10g18340	10g19510	02g03710
N6 adenine-specific (m6A) DNA methylase	0.99	8.50E-27	06g09500	06g11005	07g15310
Multifunctional methyltransferase TRM112	0.97	6.30E-09	08g14180	08g15680	04g08990
SAM methyltransferase (uncharacterized)	0.56	2.2E-73	05g08060	05g09340	08g17780
SAM methyltransferase (uncharacterized)	0.94	4.49E-45	08g14840	08g16340	04g09660
SAM methyltransferase (uncharacterized)	0.80	6.90E-39	09g16410	09g18090	03g06770

^a^ Best template modelling (TM) scores and *E*-values predicted from analyses summarized in Table S4; TM scores above 0.5 indicate proteins with similar structural folds [45]

^b^ Pfam-A family *Nol1_Nop2_Sun* was renamed *Methyltr_RsmB-F*

## Discussion

With their smaller than 3 Mbp genomes, Microsporidia from the genus *Encephalitozoon* are models of genome streamlining in parasitic eukaryotes. However, because no representative *Encephalitozoon* genome had been completely sequenced from telomere-to-telomere and since no information about their methylation states was yet available, our knowledge of the genetic/epigenetic architecture and gene content of these tiny eukaryote genomes was incomplete. To rectify this, in this study we sequenced from telomere-to-telomere the genomes of three major human-infecting *Encephalitozoon* species (*E. intestinalis*, *E. hellem* and *E. cuniculi*) and investigated their epigenetic methylation regulation capabilities using methylation data from long read sequencing platforms as well as sequence- and structure-based approaches to identify proteins involved in the corresponding processes.

DNA methylation is commonly used in eukaryotes to regulate gene expression [50] but given the sporadic distribution of methylation enzymes in fungi [16], at the onset of this study we were not sure what to expect in terms of methylation in Microsporidia. Our results strongly support the presence of 5mC and 5hmC methylation in the *Encephalitozoon* genomes with hypermethylation of the rRNA gene loci (Figs. 1 and S1). Ribosomal RNA genes are usually present in multiple copies in eukaryote genomes and their expression often silenced epigenetically by methylation at different life stages [19]. Considering that the DNA sequenced in this study originated from dormant spores, it is therefore perhaps not surprising that the rRNA genes were found hypermethylated in a DNA methylation-capable organism. Performing the same analyses on DNA isolated from biologically active meronts instead would likely result in lower methylation levels of these RNA gene loci, but further investigations will be required to determine if rRNA methylation is indeed used by *Encephalitozoon* species as a shutdown mechanism to facilitate spore survival or a byproduct caused by other metabolic activities.

Try as we might however, we could not identify the enzymes involved in the 5mC/5hmC methylation of DNA substrates in *Encephalitozoon* species using computational analyses. While many putative RNA cytosine-5 methyltransferases were found by sequence and structural homology searches (Table 2), no clear image emerged about which enzymes could act as analogs of DNMT1, DNMT3 and/or DNMT5 in *Encephalitozoon* species. In the fungal pathogen *Cryptococcus neoformans*, de novo methylases are absent and its methylation status is maintained entirely via DNMT5-mediated epigenetic inheritance [17], demonstrating that not all of these enzymes are required for maintaining DNA-methylated loci. Considering the high levels of sequence divergence in Microsporidia [51] and that nearly 25% the *Encephalitozoon* proteins could not be folded reliably (495 out of the 2075 *E. intestinalis* AlphaFold-predicted structures had pLDDT scores averaging less than 75%; Fig. S4), it is possible that one or more of these enzymes are indeed present in the genome yet remain to be identified. Alternatively, we cannot rule out a dual specificity role for the RNA methylases. RNA methylases sometimes can methylate DNA substrates, albeit with lower affinity [14, 52, 53], and this could be the case here. Although we considered the possibility of contamination by methylated rRNAs in our samples, we found no indication for such contamination in our analyses. Discarding reads smaller than 5,000 nt produced the same methylation patterns, and a thorough review of our protocols strongly argues against rRNA contamination given that the nucleic acids were isolated from transcriptionally inactive spores, that the material was thoroughly treated with RNase, that the ligation sequencing kit used for nanopore sequencing requires DNA for adapter ligation (and thus would not capture RNA) and that the PacBio platform cannot sequence RNA molecules. In any case, further in vitro work will be required to ascertain the exact roles of the *Encephalitozoon* methyltransferases predicted in this study and to identify which one(s) can act on DNA.

In a previous study, Dia and colleagues suggested that the subtelomeres in *Encephalitozoon* genomes likely serve as constitutive heterochromatin loci given their overall low coding density and flanking by rRNA genes [9]. Our DNA methylation results are congruent with this hypothesis, with the subtelomeres in *Encephalitozoon* species showing intermediate levels of DNA methylation between the hypomethylated chromosomes cores —*i.e.*, euchromatin loci— and the high levels of methylation of the rRNA genes (Figs. 1 and S1). In the ascomycete *Neurospora crassa*, both DNA methylation and heterochromatin formation loci were found to colocalize perfectly [18], and we postulate that the RNA genes in *Encephalitozoon* species act as facultative heterochromatin loci. In the fission yeast *S. pombe*, facultative heterochromatin formation of rRNA genes has been shown to be essential for cell survival during nutrient depletion by switching off energy-intensive metabolic processes [54], and the methylation of these genes in dormant *Encephalitozoon* spores is congruent with this mechanism. However, because *Encephalitozoon* species lack Dicer and Argonaute proteins [55], RNAi-dependent heterochromatin formation like in fission yeast is unlikely in these species. In *N. crassa*, both DNA methylation and heterochromatin formation were found to be independent from RNA interference [56] and, while DNA methylation is considered non-essential for heterochromatin formation in this organism [18], its presence in *Encephalitozoon* species may help facilitate heterochromatin formation in the absence of RNA interference. Considering that the presence of heterochromatin is essential to genome housekeeping [57] and that several key components including the H3K9 histone-lysine N-methyltransferase Clr4, the heterochromatin formation protein Swi6 and the FACT histone chaperone subunits Spt16/Pot3 were found encoded in the *Encephalitozoon* genomes (Table 1), H3K9me3-mediated heterochromatin formation is likely active in these organisms.

Centromeres in fungal lineages are either defined epigenetically like in the fission yeast *S. pombe* or genetically at the sequence level (point centromeres) by the presence of short, conserved DNA repeats as in the budding yeast *S. cerevisiae* [58]. In a previous study, Malik and colleagues [59] suggested that the centromeres of the microsporidium *Encephalitozoon cuniculi* were also epigenetically defined based on its retention of a few heterochromatin components that are present in *S. pombe* but absent from *S. cerevisiae*, and we believe that this is likely correct for the following reasons. In addition to heterochromatin components, the *Encephalitozoon* genomes also code for two histones H3 (Table 1), one regrouped together with histone H4 into a single genetic locus (on opposite strands) and the other located alone on a distinct chromosome. Eukaryotes with epigenetic centromeres harbor a centromere-specific histone H3 variant CENP-A [60] and, in *S. pombe*, the *cnp1* gene coding for CENP-A is segregated from other histone-related genes. In contrast, in both *S. pombe* and S*. cerevisiae*, the non-CENP-A histone H3 genes (*hht1* to *hht3*) are found adjacent to genes coding for histone H4 (*hhf1* to *hhf3*) in the exact same configurations as the *Encephalitozoon* histone H3-H4 locus. Syntenies across such a wide phylogenetic span are rare for microsporidian genomes [61], and we postulate that the standalone histone H3 in *Encephalitozoon* genomes is an ortholog of CENP-A as in *S. pombe*. Furthermore, we found no evidence for the presence of point centromeres in the *Encephalitozoon* genomes; other than the TTAGG telomere repeats, TAREs, rRNA genes, and degenerate subtelomeric repeats (illustrated by overabundant kmers; Fig. 2), no other candidate sequence was found repeated across the various chromosomes that could act in such a fashion. Considering that point centromeres are uncommon in eukaryotes (*S. cerevisiae* is an outlier even among fungi; [59]), observing a convergent evolution towards this unusual mechanism in *Encephalitozoon* spp. would have been surprising.

Although our computational analyses could not pinpoint the exact location of the centromeres in the *Encephalitozoon* genomes, they are unlikely to be in their chromosome cores. Eukaryote centromeres are usually gene poor, repeat dense and AT rich [62] yet the chromosome cores of *Encephalitozoon* genomes are gene dense, repeat poor and GC rich with little to no deviation to this pattern. In contrast, the *Encephalitozoon* (sub)telomeric regions are gene poor, repeat dense and AT rich, and thus would constitute a much better fit. Given the small sizes of their chromosomes, a (sub)telomeric location of the centromeres in *Encephalitozoon* genomes would not likely cause undue physical stress by increased pulling forces by microtubules on the kinetochores during mitosis compared to a more central location —artificial telocentric constructs in *S. cerevisiae* were shown to be mitotically stable [63]— and while uncommon, organisms with naturally occurring (sub)telocentric chromosomes do exists (*e.g.* [64, 65]). Further experimental work will be required to determine the exact location of centromeres in *Encephalitozoon* genomes. Centromeres in yeast genomes were shown to be accurately positioned from the use of Hi-C data [66] and this approach appears promising in ascertaining the position of the centromeres in *Encephalitozoon* species.

## Conclusions

As the first *Encephalitozoon* genomes sequenced from telomere-to-telomere, the data reported in this study constitute the first complete images of the genetic and epigenetic architectures of these unusually small eukaryote genomes. While our data are congruent with previous hypotheses about the sites of heterochromatin formation and the epigenetic nature of the centromeres in the *Encephalitozoon* genomes, they also raise interesting questions about the evolution of telomeres and subtelomeres in Microsporidia. Indeed, considering that the similarly sized genomes from *Ordospora* species, one of the closest known *Encephalitozoon* relatives, are estimated to code for only four rRNA gene copies despite featuring a comparable number of chromosomes [67, 68], their telomere and subtelomere architectures are bound to differ substantially. As such, future comparative studies between the two genera leveraging long read platforms and telomere-to-telomere sequencing are likely to provide interesting insights into the evolution of microsporidian genome architectures.

## Materials and methods

### Cell culture

The *Encephalitozoon* species *E. intestinalis* (ATCC 50506), *E. hellem* (ATCC 50604), and *E. cuniculi* (ATCC 50602) were obtained from the American Type Culture Collection (ATCC). *Encephalitozoon* spp. were cultured in vitro on confluent human foreskin fibroblasts (HFF) cell lines (HFF-1; ATCC SCRC-1041) in petri dishes coated with 0.1% gelatin from bovine skin. *Encephalitozoon*-infected HFF cells were maintained with 10 mL of Dulbecco’s Modified Eagle Media (DMEM) enriched with 10% fetal bovine serum (FBS) heat-inactivated for 45 min at 56˚C, 1% PSQ (100X; 12,000 Units/mL penicillin G sodium, 10,000 mg/mL streptomycin sulfate, 200 mM L-glutamine and 10 mM sodium citrate 0.14%), and 2 mM L-glutamine, and were incubated at 37˚C and 5% CO_2_. The dimethyl sulfoxide (DMSO) from the *Encephalitozoon* cultures was removed by replacing the media 24 h post-infection of the HFF cells. Henceforth, the cell culture media was renewed by replacing half of the media with fresh enriched DMEM when the media showed pH changes or when it became turbid (within 2 to 4 days). *Encephalitozoon*-infected HFF cells were passaged two weeks post-infection in a 1:8 or 1:10 ratio, following trypsinization of infected cells (trypsin 0.05%).

*Encephalitozoon* spores were harvested when the infected HFF cells reached confluence (2 weeks, approximately). The infected HFF cells were detached from the petri dish with trypsin (0.05%) and lysed by passing through a 27-gauge needle three times. Host cell debris was sieved through a 5 µm polyvinylidene difluoride (PVDF) membrane filter (Tisch Scientific, Cleves, OH, USA) and spores were recovered by centrifugation (1,500 g, 20 min). Host cell membranes were eliminated by resuspending spores in 1 mL of Tween 20 (0.3% v/v) in phosphate-buffered saline (PBS) 1X filtered with a 0.22 µm hydrophilic polyether sulfate (PES) membrane filter (Techno Plastid Products AG, Trasadingen, Switzerland), followed by washing of the spores with 10 mL of PBS (1X) three times. Spores were resuspended in 10 mL of PBS (1X) and stored at 4˚C. Host cell DNA was eliminated by treatment with 10 µL of DNase I (10 mg/mL; final concentration 250 nM) and 5 µl of MgCl_2_ (1 M; final concentration 0.5 mM) for 15 min under agitation (22 rpm). DNase I activity was halted by the addition of EDTA (final concentration 3 mM) to chelate Mg^2+^ ions, which are essential for DNase activity. Spores were collected by centrifugation (1,500 g, 20 min), washed with 1 mL of PBS (1X) six times, and resuspended in 3 mL of PBS (1X). Clean *Encephalitozoon* spore samples were stored at 4˚C.

### DNA extraction and sequencing

High molecular weight (HMW) genomic DNA (gDNA) from *Encephalitozoon* spp. spores was extracted as described previously [69] and resuspended in molecular biology grade water (Invitrogen, Waltham, MA, USA) overnight. Extracted DNA was quantified by fluorometry with the AccuGreen dsDNA High-Sensitivity (HS) kit (Biotium, Hayward, CA, USA) on a Qubit 2.0 instrument (Invitrogen, Carlsbad, CA, USA), its purity was assessed from its A_260_/A_230_ and A_260_/A_280_ absorbance ratios with a microvolume spectrophotometer (DeNovix, Wilmington, DE, USA), and its HMW was ascertained by electrophoresis on agarose gel (0.8%).

The *Encephalitozoon* spp. genomes were sequenced using short and long read high-throughput platforms as follows. Illumina paired end (151 bp) libraries were prepared using the Nextera DNA Flex kit (Illumina, San Diego, CA, USA) from 50 ng of HMW gDNA and sequenced in house on an Illumina MiniSeq instrument using mid/high-throughput cartridges. Oxford Nanopore DNA libraries were prepared using the SQK-LSK109 ligation sequencing kit (Oxford Nanopore, Oxford, UK) from 500 ng of HMW gDNA pre-fragmented by centrifugation (*E. intestinalis*) with a g-TUBE (Covaris, Woburn, MA, USA) or by needle shearing (*E. hellem/E. cuniculi*) with a 27-gauge needle (Becton Dickinson, Franklin Lakes, NJ, USA) to increase sequencing yields. Oxford Nanopore DNA libraries were sequenced in house using R.9.4.1 flow cells (FLO-MIN06D) on a MinION Mk1B instrument. The *E. intestinalis* ATCC 50506 genome was also sequenced with PacBio using the SMRTbell Express Template Prep Kit 2.0 (Pacific Biosciences, Menlo Park, CA, USA) and the SMRT Cell 1 M v3 LR on a Sequel II instrument at the Cold Spring Harbor Laboratory (CSHL; Cold Spring Harbor, NY, USA).

### Genome assembly

Nanopore raw FAST5 datasets were basecalled and converted to FASTQ format post-sequencing with Guppy v3.2.1 (*E. intestinalis/E. hellem*) and v4.0.15 (*E. cuniculi*) (Oxford Nanopore, Oxford, UK). Dataset metrics and read length distributions were calculated and plotted from the FASTQ files with read_len_plot.py. Nanopore FASTQ datasets were assembled with Flye [70] v2.5 (*E. intestinalis/E. hellem*) and v2.8.2 (*E. cuniculi*) using the '–nano-raw', '–asm-coverage 200' and '–genome-size 3.0 m' command line switches. The lack of contaminants in the resulting assemblies was ascertained by BLAST homology searches [71]. Consensus sequences were improved by mapping long read then short read data onto the assemblies. Long read-based corrections for *E. intestinalis/E. hellem* and *E. cuniculi* were performed with Nanopolish v0.11.1 (https://github.com/jts/nanopolish) and Medaka v1.2.3 (https://github.com/nanoporetech/medaka), respectively. Illumina read datasets were assessed with FASTQC v0.11.7 (https://www.bioinformatics.babraham.ac.uk/projects/fastqc/), Nextera adapters were removed with Cutadapt v2.4 [72] (*E. intestinalis/E. hellem*) and Fastp v0.20.1 [73] (*E. cuniculi*), and nanopore-corrected assemblies were further corrected with Illumina data iteratively using Pilon v1.22 [74] as implemented in run_pilon.pl until no more improvement was detected. Assembly metrics were calculated with QUAST 5.0.2 [75] from the polished consensus sequences. Chromosome completeness was investigated by searching for the presence of telomere repeat units on both chromosome ends with check_for_telomeres.pl v0.3a.

### Genome annotation

The *Encephalitozoon* genomes were annotated with Apollo v2.5.0 [76] and the A2GB pipeline (https://github.com/PombertLab/A2GB) as described below. Protein-coding genes, ribosomal RNAs and transfer RNAs were predicted with Prodigal v2.6.3 [77], RNAmmer v1.2 [78] and tRNAscan-SE v2.0.4 [79], respectively. RNAmmer and tRNAscan-SE outputs were converted to GFF format with RNAmmer_to_GFF3.pl and tRNAscan_to_GFF3.pl from A2GB, respectively, and predicted genes were loaded as separate tracks into Apollo using its built-in tools. BLASTN and TBLASTN homology searches [71] were performed against the newly sequenced *Encephalitozoon* genomes using genes and proteins from previously annotated *Encephalitozoon* genomes [7, 8] from the NCBI Reference Sequence Database (RefSeq accession numbers GCF_000146465.1, GCF_000277815.2, GCF_000280035.1, GCF_000091225.1). BLAST tabular outputs (–outfmt 6) were converted to GFF format with BLAST_to_GFF3.pl from A2GB and loaded as independent tracks with Apollo’s built-in tools. Preliminary gene annotations were created from the information contained within the Apollo tracks and exported in GFF3 format using Apollo’s embedded tools for curation with A2GB. Briefly, GFF3 files were converted to EMBL format with ApolloGFF3toEMBL.pl, and introns were manually added to the *Encephalitozoon* annotations with Artemis v18.1.0 [80]. Missing start methionines and stop codons were searched for in the EMBL files with check_problems.pl from A2GB. The *E. cuniculi* ATCC 50602 genotype (genotype III) was inferred by mapping the internal transcribed spacer (ITS) in-between its small and large subunit (SSU and LSU) rRNA genes against other known genotypes as described previously [69].

Protein functions were inferred using sequence- and structure-based homology approaches. Sequence homology searches were performed with InterProScan5 v5.46–81.0 [81] and with DIAMOND v2.0.4 [82] against UniProt’s SwissProt/TrEMBL databases [83] and against *Encephalitozoon* RefSeq protein datasets. *E. intestinalis* protein structures were predicted with AlphaFold v2.0 [84] and RaptorX v1.66 [85] using default settings from 3DFI v0.5 [86]. *E. hellem* and *E. cuniculi* subtelomere protein structures were predicted with AlphaFold and RaptorX; structures from *E. intestinalis* were used as proxies for *E. hellem*/*E. cuniculi* orthologs found in their chromosome cores. Confidence scores for predicted structures were independently assessed with VoroCNN [87]. AlphaFold (predicted local distance difference test; pLDDT) and VoroCNN protein folding confidence scores were plotted with make_score_distributions.py. Structural homology searches were performed with GESAMT v7.1 [88] against experimental proteins from the RCSB Protein Data Bank [89] as implemented in 3DFI. Protein functions were inferred from these analyses with curate_annotations.pl from A2GB. Accession numbers were generated as described in A2GB with NCBI’s TBL2ASN v25.8 (https://www.ncbi.nlm.nih.gov/genbank/tbl2asn2/). Protein annotation completeness was assessed with BUSCO 5.3.0 [90] and sequencing, assembly and annotation metrics were aggregated with MultiQC v1.12 ([91]; Additional data S5). Transmembrane proteins were predicted with DeepTMHMM v1.0.11 [92].

### Methylation analyses

Methylated bases in the *Encephalitozoon* genomes were inferred from the nanopore sequencing datasets with Megalodon v2.4.1 (https://github.com/nanoporetech/megalodon) and Tombo v1.5.1 [93]. Megalodon methylation inferences were performed using the high accuracy model from Guppy v6.0.1 (dna_r9.4.1_450bps_hac.cfg) and the R9.4.1 remora base model (dna_r9.4.1_e8) on an RTX A4000 graphics processing unit (GPU) (NVIDIA, Santa Clara, CA, USA). Tombo methylation inferences were generated from a total of 500 K reads per *Encephalitozoon* genome briefly as follows (details are provided in Additional data S2). Raw FAST5 datasets were basecalled with Guppy v6.0.1 on an NVIDIA RTX A4000 GPU and the basecalled reads converted from multi to single FAST5 datasets with multi_to_single_fast5 from the Megalodon v2.4.1 package. Basecalled reads were mapped onto the genomes, base modifications were detected, and plots were generated with Tombo’s 'resquiggle', 'detect_modifications' and 'plot most_significant' commands, respectively. Sequences flanking genome loci with high proportions of methylated bases were exported with Tombo’s 'text_output signif_sequence_context' command and motifs present therein detected with the MEME suite v5.4.1 [94]. Methylation sites were visualized from the mapped BAM files with IGV v2.11.7 [95] using a minimum base modification probability of 0.8. Relative distributions of 5mC and 5hmC bases were calculated from the BED files generated with Megalodon using methyldib.pl v0.1 and were plotted with Circos v0.69–9 [96].

Methylated 4mC and 6mA sites in the *E. intestinalis* genome were independently inferred from its PacBio continuous long read (CLR) sequencing dataset with the Base Modification Analysis protocol from SMRT Link v10.2 (Pacific Biosciences, Menlo Park, CA, USA) using default parameters whereas 5mC sites were further investigated as follows. PacBio CLR reads were converted to HiFi circular consensus sequence (CCS) reads with ccs v6.4.0 (https://github.com/PacificBiosciences/ccs), 5mC bases in the HiFi CCS reads were inferred with primrose v1.3.0 (https://github.com/PacificBiosciences/primrose), the 5mC-tagged HiFi CCS reads were mapped onto the *E. intestinalis* genome with pbmm2 v1.9.0 (https://github.com/PacificBiosciences/pbmm2), and methylated CpG sites were inferred from this alignment with pb-CpG-tools v1.1.0 (https://github.com/PacificBiosciences/pb-CpG-tools).

To rule out contamination from methylated rRNAs in the datasets, the BAM files generated with megalodon were filtered by size to keep only sequencing reads of at least 5,000 nt (-m 5000) with the view function from samtools v1.16.1 [97], then visualized again with IGV. Sequencing depths at the rRNA gene loci were also compared to the overall sequencing depths of the *Encephalitozoon* genomes by read mapping onto these genomes with minimap2 v2.24 [98] as implemented in get_SNPs.pl v2.0e, and the overall metrics and length distributions of the sequencing reads covering the rRNA gene loci were further compared to the full datasets by extracting the reads overlapping with the rRNA gene loci from the BAM files with samtools (see Additional data S2 for details) followed by plotting with read_len_plot.py.

### Nucleotide biases, tandem repeats, chromosome partitioning, and synteny analyses

Nucleotide biases of the *Encephalitozoon* genomes were profiled with nucleotide_biases.pl using sliding windows of 1,000 nt. Tandem repeats in *Encephalitozoon* genomes were identified with Tandem Repeat Finder [99] v4.09 (match weight 2; mismatch penalty 5; indel penalty 7, match probability 80; indel probability 10; min score 50; max period 2000; max tr length 1) and with TideHunter v1.5.3 [100], the latter using default parameters expect for a minimum length of 5 bp ('-m 5') to include telomere repeat units. Repetitive elements (represented by overabundances of kmers) were further searched for with k_counter.py using sliding windows of 1,000 nt and kmer values from 5 to 10 using the following formula: 1 – (number of unique kmers/number of possible kmers per sliding window). Kmers were then plotted with k_plotter.py. Longer repeated/duplicated loci in *Encephalitozoon* genomes were searched for with BLASTN homology searches using each genome (query) against itself (subject), and the results (in –outfmt 6 format) were parsed with b2links.pl (minimum bitscores and lengths of 5,000 and 1,000, respectively) for plotting with Circos v0.69–9 [96]. Chromosome cores in *Encephalitozoon* spp. were hereby defined as the regions encompassing the center of all orthologous chromosomes, whose GC-contents decrease from center to edges [6, 69], the latter of which are flanked by abrupt shifts in GC contents [9]. Subtelomeres were defined as the regions starting from the rRNA genes to the chromosome cores. The position of the telomeres, subtelomeres, and chromosome cores were delineated with chrom_table.pl v0.3 using the tab-delimited output files from TideHunter, the tab-delimited (.tsv) file containing the lower GC points as identified with gc_plot.pl v0.3 using sliding windows of 2,500 nt, the genome (.fasta) file to calculate chromosome lengths, and the GenBank (.gb) file to account for the number of genetic features (rRNAs, tRNAs, CDSs, core/subtelomere genes) per chromosome. Subtelomere proteins in the three *Encephalitozoon* spp. were extracted from their GenBank (.gb) annotations with get_sub_proteins.pl v0.2 and the corresponding data was summarized into a master table (Table S1) with subtel_table.pl v0.2. Dot plot comparisons within and between *Encephalitozoon* genomes were performed with D-GENIES v1.3.1 [101]. Gene clusters conserved across the three *Encephalitozoon* species were inferred with run_syny.pl v0.5.2 from SYNY (https://github.com/PombertLab/SYNY) using default parameters.

### Homology searches against *Encephalitozoon* data

TBLASTN and BLASTP sequence homology searches against the *Encephalitozoon* genomes and proteins, respectively, were performed with the NCBI BLAST + v2.12.0 suite [71]. Pfam Hidden Markov models (v2021-11–15) were searched against the *Encephalitozoon* protein sequences with hmmsearch from HMMER v3.3.2 [102], and motifs of interest were investigated with regular expressions using parse_pfam_search.pl v0.1. Conserved domains in *Encephalitozoon* proteins were further searched for with NCBI’s batch CD-search against its conserved domain database (CDD) [103], then parsed by regular expressions with parse_cd_search.pl v0.1. Experimental and predicted proteins of interest were downloaded from the RCSB Protein Data Bank [89] and the AlphaFold-EBI protein structure database [104], respectively, then searched against the *Encephalitozoon* AlphaFold and RaptorX predicted structures with GESAMT v7.1 [88] and Foldseek v3-915ef7d [105], using run_GESAMT.pl and run_foldseek.pl from 3DFI [86]. Predicted local distance difference test (pLDDT) confidence scores in AlphaFold-EBI structures were assessed with av_pLDDT_from_pdb.pl v0.1.

### Gene ontology searches

Proteins involved in telomere maintenance (GO:0,000,723), heterochromatin formation (GO:0,031,507), centromere complex assembly (GO:0,034,508), and methylation (GO:0,032,259) in *Encephalitozoon* species were searched for independently using data retrieved from PomBase [106] and from UniProtKB [83].

*Schizosaccharomyces pombe* protein sequences from selected gene ontologies and descendant processes were downloaded from PomBase and their predicted tridimensional structures were downloaded from the AlphaFold-EBI protein structure database [104] using the links provided in PomBase. *S. pombe* sequence homologs in *Encephalitozoon* spp. were searched for with BLASTP v2.12.0 + [107] with an *E*-value cutoff of 1e-05 whereas structural homologs were searched for with FoldSeek v3-915ef7d [105] using the 3Di + AA Gotoh-Smith-Waterman scoring scheme as implemented in run_foldseek.pl from 3DFI v1.0.1a [86]. TM-scores for candidate matches were calculated with MICAN-SQ v2019-11–27 [108] and summarized with pombase_matches.pl v0.2.

Gene ontology inferences derived from UniProtKB data were performed with the QueGO pipeline (https://github.com/PombertLab/QueGO). QueGO (Query Gene Ontology) is a UniProtKB scrapper that returns experimentally verified protein sequences and structures related to the queried gene ontology (GO) terms and/or keywords. It then performs structural homology searches against a provided set of protein structures. Relevant GO terms were identified from the Gene Ontology Consortium metadata (http://purl.obolibrary.org/obo/go.obo) and corresponding UniProt data were retrieved with run_QueGO.pl v0.5f using the '-v' (experimentally verified), '-m' (method) X-ray, and '-g' (go terms/keywords) command line switches with the following terms/keywords: adhesion, antigen binding, autophagy, entry into host, heterochromatin, host cell surface binding, centromere, epigenetic, methylation, symbiont, and telomere. Structural homologs of the keyword-specific structures were searched for in the *Encephalitozoon* predicted protein structures using GESAMT v7.1 [88] and FoldSeek v3-915ef7d [105] as implemented in run_QueGO.pl v0.8.4 (https://github.com/PombertLab/QueGO).

### Protein–protein docking

Putative protein–protein interactions were predicted with Megadock v4.1.4 [109] as implemented in dockit.pl v0.2. Briefly, to reduce noise from improperly folded proteins and to reduce computation time, only the top ranked AlphaFold-predicted models from each *E. intestinalis* protein (as described earlier in genome annotation) and showing average pLDDT scores of at least 75% were selected (with get_top_models.pl v0.1) for protein–protein docking simulations. Known homo- and hetero-protein complexes in *E. intestinalis* were further predicted with AlphaFold-Multimer [110] from AlphaFold v2.2 and selected by their pLDDT scores. Molecular docking inferences were performed with megadock-gpu on an NVIDIA RTX A6000 using the proteins of interest as receptors, all top ranked AlphaFold-predicted *E. intestinalis* proteins as ligands (min pLDDT = 75), 3 predictions per rotation and a total of 10,000 output predictions. Protein–protein interaction (PPI) scores were calculated with ppiscore (Megadock) and protein structures for the top binding partners were generated with decoygen (Megadock) as implemented in dockit.pl. PPI structures generated (in PDB format) were visualized with ChimeraX v1.4 [111].

## Supplementary Information

**Additional file 1.**

**Additional file 2:** **Figure S1.** Physical and methylation maps of the *Encephalitozoon hellem* ATCC 50604 and *Encephalitozoon cuniculi* ATCC 50602 genomes. From outer to inner concentric rings: 1) AT and GC nucleotide biases (grey and red lines, respectively); 2) GT and AC nucleotide biases (blue and green lines, respectively); 3) GA and CT nucleotide biases (purple and yellow lines, respectively); 4 and 5) relative proportions of 5hmC (blue) and 5mC (red) methylated sites across each chromosome. Repeated loci between chromosomes (in grey) and within chromosomes (color-coded per chromosome) are highlighted by ribbons in the center of the concentric circles. **Figure S2.** Dot plot comparisons between *E. intestinalis* and other *Encephalitozoon* genomes. Chromosome numbers I to XI are represented by Arabic numerals 01 to 11. For, *E. cuniculi* ATCC 50602, the contig (cg) number is also indicated between parentheses. Dot plots generated with D-GENIES were composited and cleaned up with Adobe Illustrator. **Figure S3.** Chromosomal reorganizations between *Encephalitozoon* genomes. The *E. intestinalis*, *E. hellem* and *E. cuniculi* chromosomes are indicated by the letter i, h and c, respectively, followed by their chromosome number in Arabic numerals. Relocations between the *E. intestinalis*/*E. hellem*, *E. intestinalis*/*E. cuniculi* and *E. hellem*/*E. cuniculi* chromosomes are highlighted by purple, magenta and cyan ribbons, respectively. Syntenic regions are highlighted by gray ribbons. GC percentage plots are inserted in-between the chromosome representations and their corresponding ribbons. **Figure S4.** Distributions of quality scores for the *E. intestinalis* predicted protein structures. A. Distributions of the predicted Local Distance Difference Test (pLDDT) averaged scores for the known and hypothetical proteins predicted with AlphaFold. B. Distributions of the voroCNN confidence scores for the AlphaFold and RaptorX predicted structures. **Figure S5.** Location of the gene coding for Sirtuin 2 in *Encephalitozoon* genomes. Locus tags for each gene are indicated inside the corresponding boxes. Except for Sirtuin 2 missing from *E. intestinalis*, this cluster is perfectly conserved across *Encephalitozoon* genomes. The gene coding for Sirtuin 2 was not found anywhere in the *E. intestinalis* genome.

**Additional file 3: Table S1.** Chromosome lengths, partitions, and features distributions. **Table S2.** Example of telomere adjacent repeats (TARE) found in the *Encephalitozoon* chromosomes. **Table S3.** Subtelomere proteins shared between human-infecting *Encephalitozoon* spp. **Table S4.** Summary of computational predictions used to infer proteins listed in Tables 1 and 2.

## Data Availability

The genomes of the *Encephalitozoon* species *E. intestinalis* ATCC 50506, *E. hellem* ATCC 50604 and *E. cuniculi* ATCC 50602 were deposited in NCBI under accession numbers CP075158 to CP075168, CP075147 to CP075157 and CP091431 to CP091441, respectively. Sequencing data were deposited in the NCBI Sequence Read Archive as follows. *E. intestinalis* data were deposited under accessions SRR17865591 (Illumina), SRR17865590 (Nanopore) and SRR17865589 (PacBio). *E. hellem* data were deposited under accessions SRR17853475 (Illumina) and SRR17853474 (Nanopore). *E. cuniculi* data were deposited under accessions SRR17858635/SRR17858636 (Illumina) and SRR17858634 (Nanopore). Additional data files (Data S1 – S6) and other miscellaneous large data files generated as part of this manuscript (*e.g.*, BAM files) are available on Zenodo (https://doi.org/10.5281/zenodo.7415325). Custom scripts written as part of this manuscript are freely available on GitHub (https://github.com/PombertLab) and included as a single archive (Additional data S6) on Zenodo.

## Abbreviations

4mC: 4-Methylcytosine
5hmC: 5-Hemimethylcytosine
5mC: 5-Methylcytosine
6mA: 6-Methyladenine
ATCC: American type culture collection
ATP: Adenosine triphosphate
CCS: Circular consensus sequence
CDS: Coding sequence
CLR: Continuous long read
CLRC: Clr4 methyltransferase complex
CST: Cdc13-Stn1-Ten1
DDB1: DNA damage binding protein 1
DMEM: Dulbecco’s modified eagle media
DMSO: Dimethyl sulfoxide
DNMTs: DNA methyltransferases
Dos: Delocalization of Swi6
EDTA: Ethylenediaminetetraacetic acid
FACT: Facilitates chromatin transcription
FBS: Fetal bovine serum
FISH: Fluorescence in situ hybridization
gDNA: Genomic DNA
GO: Gene ontology
GPU: Graphics processing unit
H3K9me: Histidine 3 lysine 9 methylation
H4K16: Histone H4 Lysine 16
HFF: Human foreskin fibroblasts
HMW: High molecular weight
HS: High sensitivity
ITS: Internal transcribed spacer
LSU: Large subunit
ORF: Open reading frame
Orp1/ORC1: Origin recognition complex protein 1
PBS: Phosphate buffered saline
PES: Hydrophilic polyether sulfate
pLDDT: Predicted local distance difference test
PPI: Protein–protein interaction
PSQ: 100X; 12,000 Units/mL penicillin G sodium, 10,000 mg/mL streptomycin sulfate, 200 mM L-glutamine and 10 mM sodium citrate 0.14%
PVDF: Polyvinylidene difluoride
QueGO: Query gene ontology
RITS: RNA-induced initiation of transcriptional silencing
RNAi: RNA interference
rRNA: Ribosomal RNA
SAM: S-adenosyl-L methionine
SIR: Silent information regulator
siRNAs: Small interfering RNAs
SSU: Small subunit
TAREs: Telomere-associated repeat elements
tRNA: Transfer RNA
Trt1/TERT: Telomerase reverse transcriptase
UBM: Unknown base modification
