# Supplementary material for: Telomere-to-Telomere genome assemblies of human-infecting Encephalitozoon species
Source: BMC Genomics. 2023 May 4;24:237. doi: 10.1186/s12864-023-09331-3 (PMC10158259; doi:10.1186/s12864-023-09331-3)
Supplement: Supplementary file 1 — Additional file 1. [file 12864_2023_9331_MOESM1_ESM.pdf]

Supplementary data files S1 to S6

Supplementary data files S1 to S6 and other related large files produced as part of this manuscript are available on Zenodo: https://doi.org/10.5281/zenodo.7415324.

Description – *Data S1 to S6*

Supplementary Data 1 - Data_S1_repeats.zip

Data files containing the results from TideHunter and Tandem Repeat Finder analyses

Supplementary Data 2 - Data_S2_methylation_analyses.zip

Small data files and examples of command lines used for Megalodon, Tombo and Primrose analyses. Large BAM files are provided in separate archives (Methylation_*.tar.gz)

Supplementary Data 3 - Data_S3_Homology_searches.zip

Results from miscellaneous homology searches

Supplementary Data 4 - Data_S4_PPIscores.zip

Results from protein-protein interactions (PPI) analyses with MegaDock; includes PPIscores between histones and methyltransferases (ppiscores_histones_vs_methylases.tsv) and between histones and all proteins with average alphafold pLDDT scores >= 75 (ppiscores_histones_vs_all.tsv). All files used for computation are provided in a separate archive (megadock.tar.gz)

Supplementary Data 5 - Data_S5_MultiQC_report.zip

Sequencing, assembly and annotation metrics aggregated with MultiQC v1.12

Supplementary Data 6 - Data_S6_scripts.zip

Code written as part this manuscript

Description – *Miscellaneous large data files*

Methylation_E_intestinalis_50506.tar.gz

Methylation_E_hellem_50604.tar.gz

Methylation_E_cuniculi_50602.tar.gz

Data archives containing BAM files and miscellaneous files for Megalodon and Tombo methylation analyses

Protein_stuctures_E_intestinalis_50506.tar.gz

Protein_stuctures_E_hellem_50604.tar.gz

Protein_stuctures_E_cuniculi_50602.tar.gz

Predicted AlphaFold and/or RaptorX protein structures. *E. intestinalis* (all proteins); *E. hellem/E. cuniculi* (subtelomeric proteins).

megadock.tar.gz

Includes all files used to perform molecular docking with Megadock

kmers_analysis_Encephalitozoons.tar.gz

Archive containing the data and figures for sliding window kmer analyses
