# Supplementary material for: Telomere-to-Telomere genome assemblies of human-infecting Encephalitozoon species
Source: BMC Genomics. 2023 May 4;24:237. doi: 10.1186/s12864-023-09331-3 (PMC10158259; doi:10.1186/s12864-023-09331-3)
Supplement: Supplementary file 2 — Additional file 2: Figure S1. Physical and methylation maps of the Encephalitozoon hellem ATCC 50604 and Encephalitozoon cuniculi ATCC 50602 genomes. From outer to inner concentric rings: 1) AT and GC nucleotide biases (grey and red lines, respectively); 2) GT and AC nucleotide biases (blue and green lines, respectively); 3) GA and CT nucleotide biases (purple and yellow lines, respectively); 4 and 5) relative proportions of 5hmC (blue) and 5mC (red) methylated sites across each chromosome. Repeated loci between chromosomes (in grey) and within chromosomes (color-coded per chromosome) are highlighted by ribbons in the center of the concentric circles. Figure S2. Dot plot comparisons between E. intestinalis and other Encephalitozoon genomes. Chromosome numbers I to XI are represented by Arabic numerals 01 to 11. For, E. cuniculi ATCC 50602, the contig (cg) number is also indicated between parentheses. Dot plots generated with D-GENIES were composited and cleaned up with Adobe Illustrator. Figure S3. Chromosomal reorganizations between Encephalitozoon genomes. The E. intestinalis, E. hellem and E. cuniculi chromosomes are indicated by the letter i, h and c, respectively, followed by their chromosome number in Arabic numerals. Relocations between the E. intestinalis/E. hellem, E. intestinalis/E. cuniculi and E. hellem/E. cuniculi chromosomes are highlighted by purple, magenta and cyan ribbons, respectively. Syntenic regions are highlighted by gray ribbons. GC percentage plots are inserted in-between the chromosome representations and their corresponding ribbons. Figure S4. Distributions of quality scores for the E. intestinalis predicted protein structures. A. Distributions of the predicted Local Distance Difference Test (pLDDT) averaged scores for the known and hypothetical proteins predicted with AlphaFold. B. Distributions of the voroCNN confidence scores for the AlphaFold and RaptorX predicted structures. Figure S5. Location of the gene coding for Sirtuin 2 in Ence [file 12864_2023_9331_MOESM2_ESM.pdf]

Figure S1: Physical and methylation maps of the *Encephalitozoon hellem* ATCC 50604 and *Encephalitozoon cuniculi* ATCC 50602 genomes. From outer to inner concentric rings: 1) AT and GC nucleotide biases (grey and red lines, respectively); 2) GT and AC nucleotide biases (blue and green lines, respectively); 3) GA and CT nucleotide biases (purple and yellow lines, respectively); 4 and 5) relative proportions of 5hmC (blue) and 5mC (red) methylated sites across each chromosome. Repeated loci between chromosomes (in grey) and within chromosomes (color-coded per chromosome) are highlighted by ribbons in the center of the concentric circles.

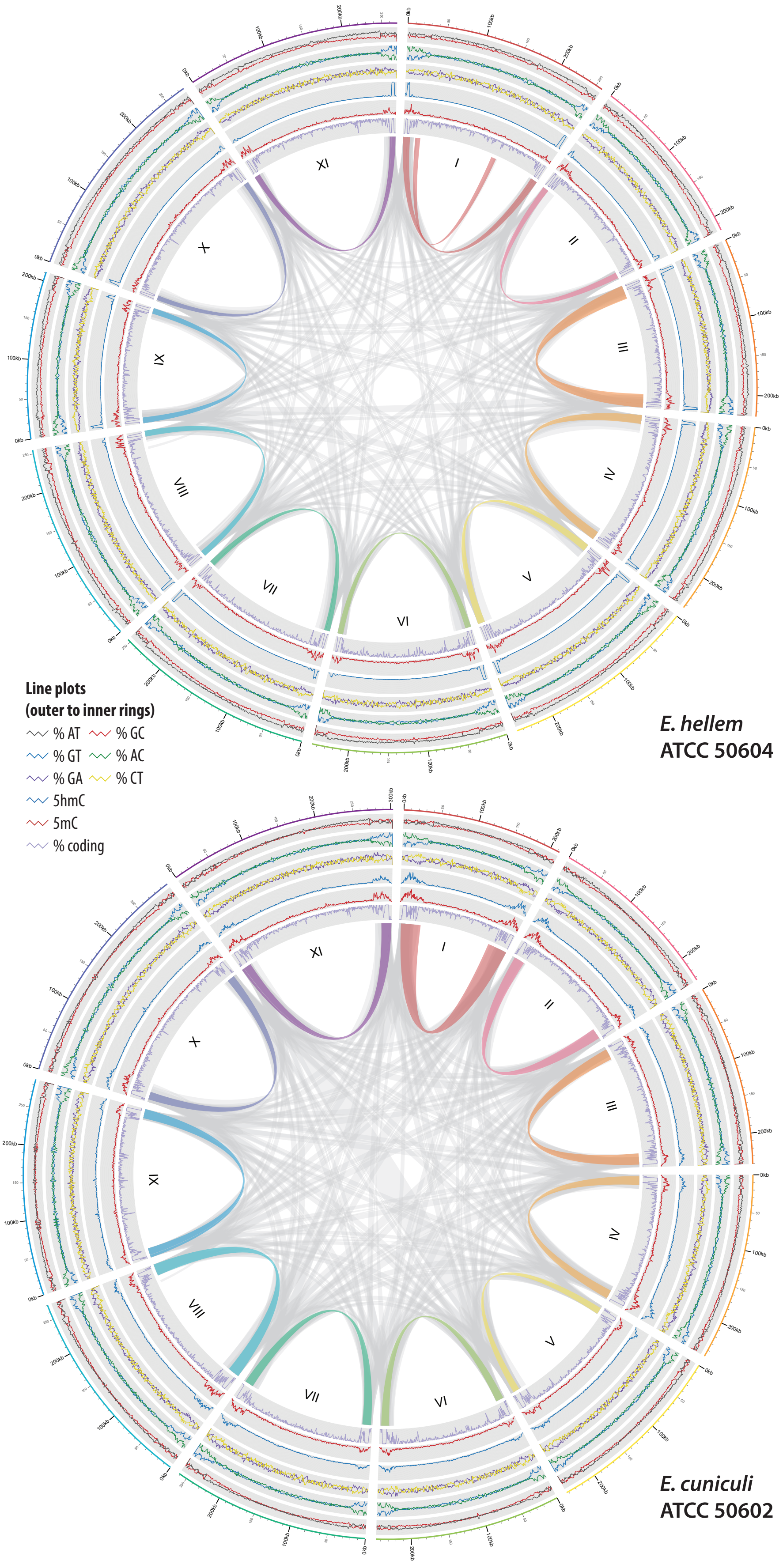

Supplementary Figure 1

Figure S2. Dot plot comparisons between *E. intestinalis* and other *Encephalitozoon* genomes. Chromosome numbers I to XI are represented by Arabic numerals 01 to 11. For, *E. cuniculi* ATCC 50602, the contig (cg) number is also indicated between parentheses. Dot plots generated with D-GENIES were composited and cleaned up with Adobe Illustrator.

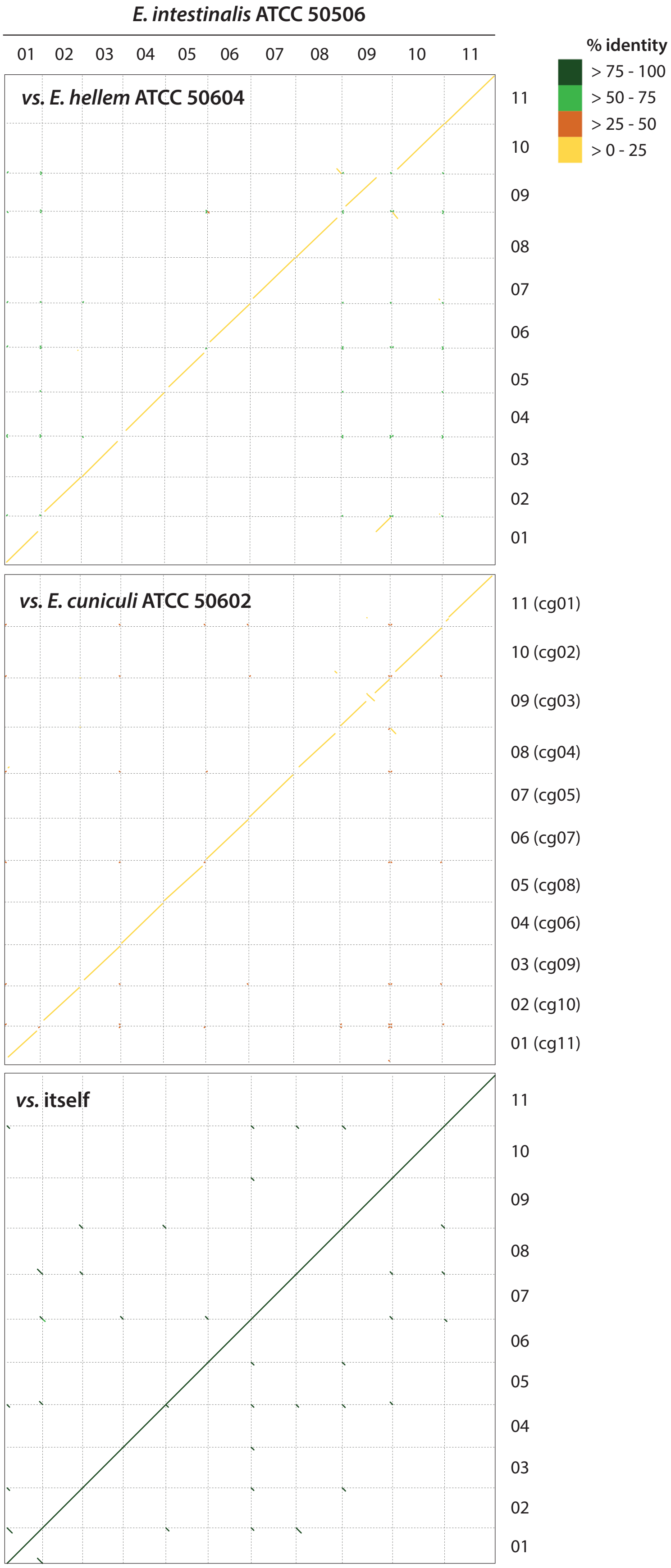

Supplementary Figure 2

Figure S3. Chromosomal reorganizations between *Encephalitozoon* genomes. The *E. intestinalis*, *E. hellem* and *E. cuniculi* chromosomes are indicated by the letter i, h and c, respectively, followed by their chromosome number in Arabic numerals. Relocations between the *E. intestinalis/E. hellem*, *E. intestinalis/E. cuniculi* and *E. hellem/E. cuniculi* chromosomes are highlighted by purple, magenta and cyan ribbons, respectively. Syntenic regions are highlighted by gray ribbons. GC percentage plots are inserted in-between the chromosome representations and their corresponding ribbons.

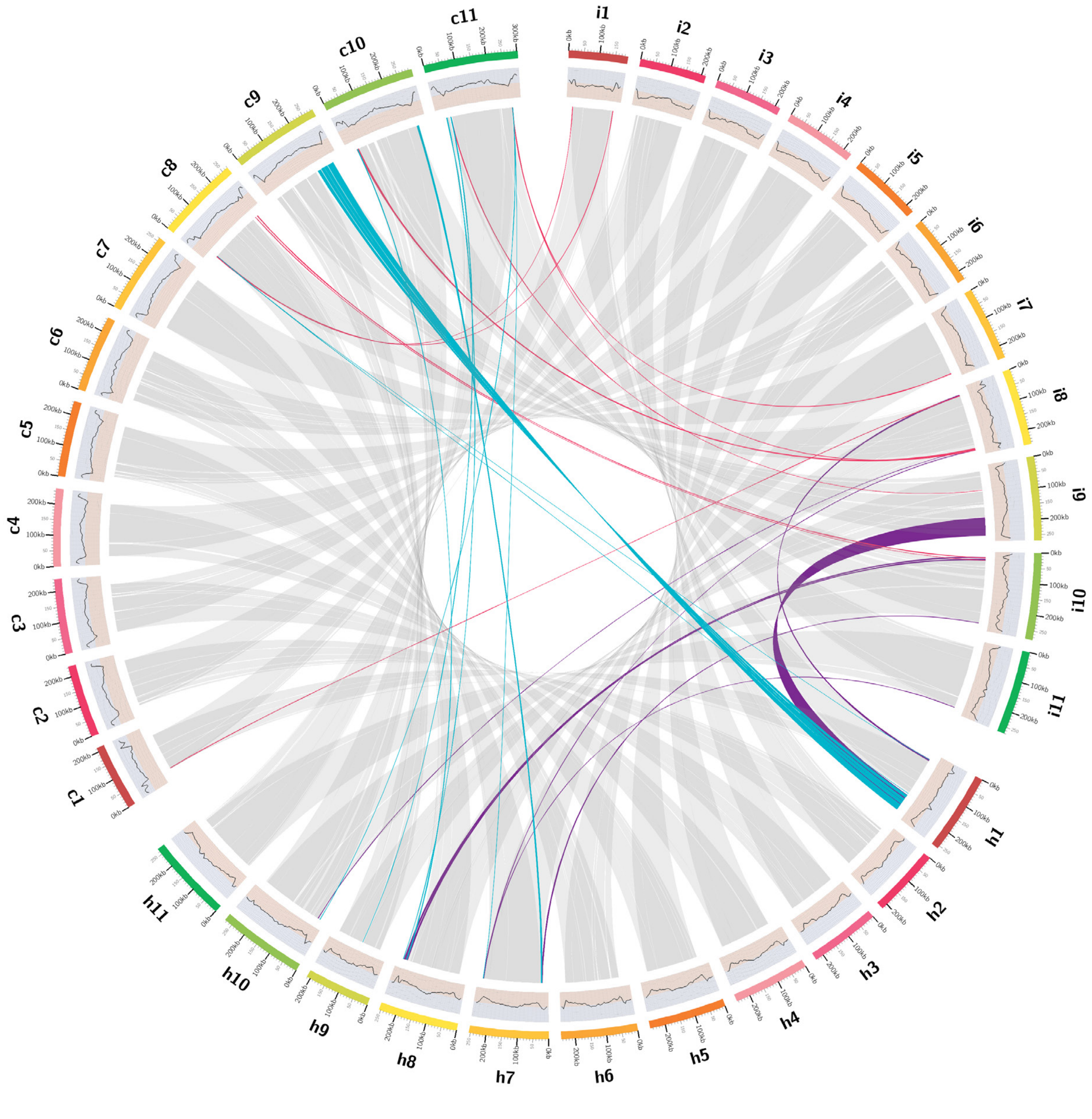

Supplementary Figure 3

Figure S4. Distributions of quality scores for the *E. intestinalis* predicted protein structures. A. Distributions of the predicted Local Distance Difference Test (pLDDT) averaged scores for the known and hypothetical proteins predicted with AlphaFold. B. Distributions of the voroCNN confidence scores for the AlphaFold and RaptorX predicted structures.

A. pLDDTs

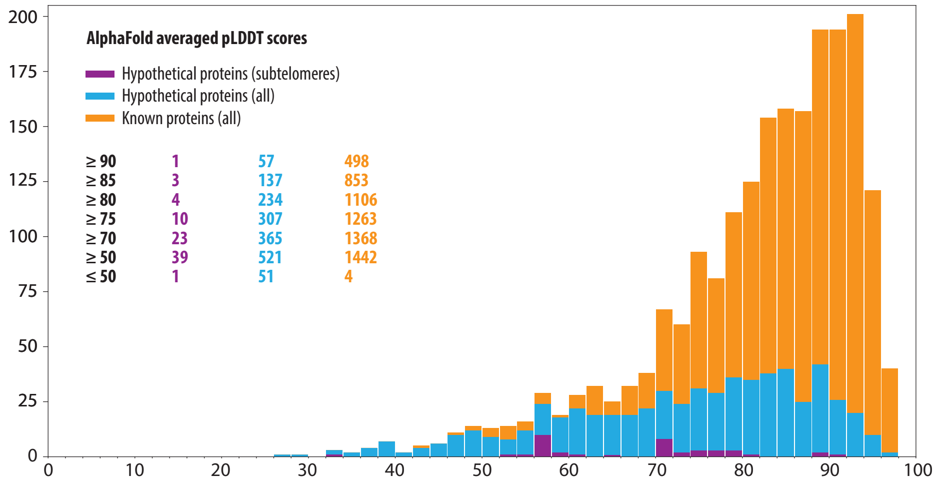

B. VoroCNN scores

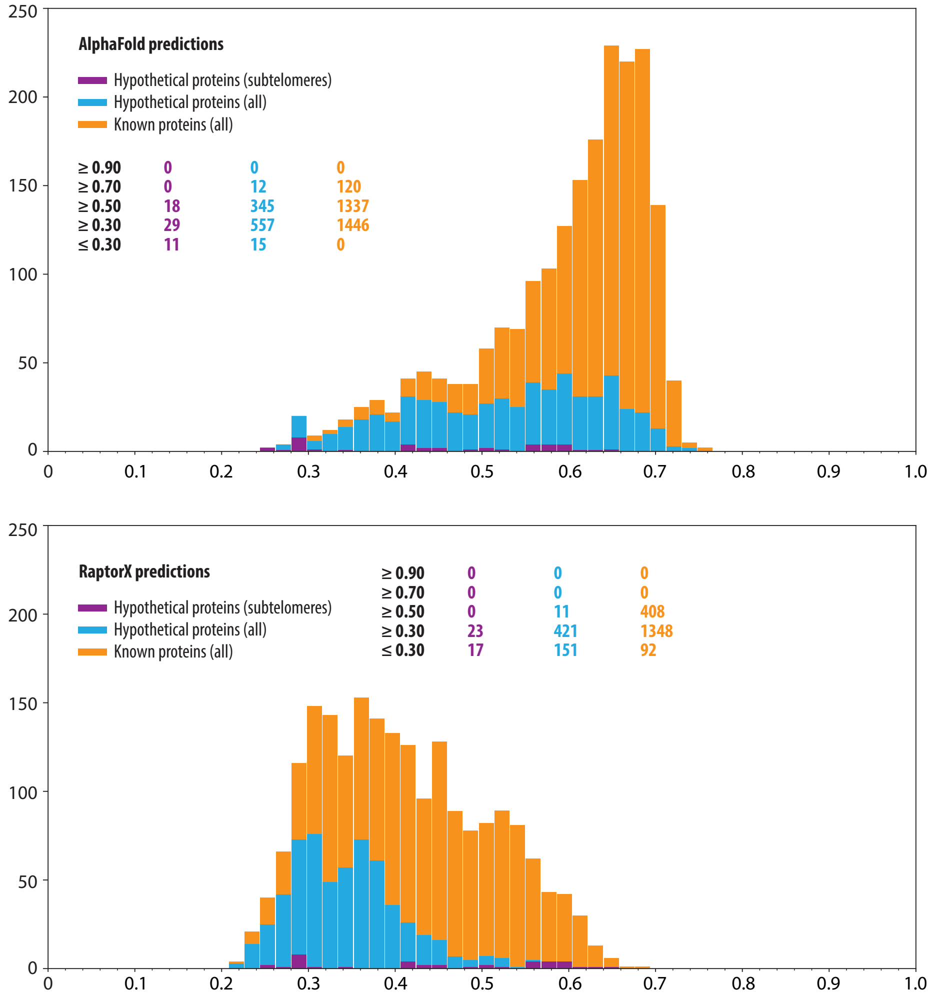

Supplementary Figure 4

Figure S5. Location of the gene coding for Sirtuin 2 in *Encephalitozoon* genomes. Locus tags for each gene are indicated inside the corresponding boxes. Except for Sirtuin 2 missing from *E. intestinalis*, this cluster is perfectly conserved across *Encephalitozoon* genomes. The gene coding for Sirtuin 2 was not found anywhere in the *E. intestinalis* genome.

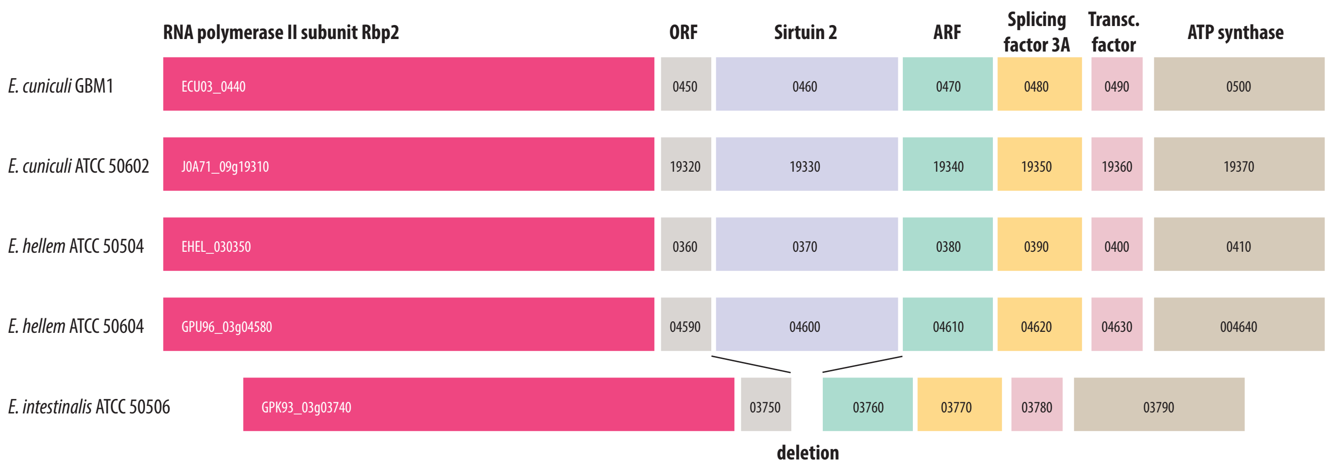

Supplementary Figure 5
